# Ultraviolet-B Irradiation Increases Antioxidant Capacity of Pakchoi (*Brassica rapa* L.) by Inducing Flavonoid Biosynthesis

**DOI:** 10.3390/plants11060766

**Published:** 2022-03-13

**Authors:** Juan Hao, Panpan Lou, Yidie Han, Lijun Zheng, Jiangjie Lu, Zhehao Chen, Jun Ni, Yanjun Yang, Maojun Xu

**Affiliations:** 1 Zhejiang Provincial Key Laboratory for Genetic Improvement and Quality Control of Medicinal Plants, Hangzhou Normal University, Hangzhou 311121, China; juanhao@hznu.edu.cn (J.H.); 2019111010029@stu.hznu.edu.cn (P.L.); 2020111010057@stu.hznu.edu.cn (Y.H.); 2021111010056@stu.hznu.edu.cn (L.Z.); lujj@hznu.edu.cn (J.L.); zhchen@hznu.edu.cn (Z.C.); nijun@hznu.edu.cn (J.N.); yjyang@hznu.edu.cn (Y.Y.); 2 Key Laboratory of Hangzhou City for Quality and Safety of Agricultural Products, College of Life and Environmental Sciences, Hangzhou Normal University, Hangzhou 311121, China

**Keywords:** pakchoi, greenhouse, UV-B, antioxidant activity, flavonoids, biosynthetic pathway

## Abstract

As an important abiotic stress factor, ultraviolet-B (UV-B) light can stimulate the accumulation of antioxidants in plants. In this study, the possibility of enhancing antioxidant capacity in pakchoi (*Brassica rapa* L.) by UV-B supplementation was assessed. Irradiation with 4 µmol·m^−2^·s^−1^ UV-B for 4 h or 2 µmol·m^−2^·s^−1^ UV-B for 24 h significantly increased the 1,1–diphenyl–2–picrylhydrazyl (DPPH) scavenging activity and total reductive capacity, as a result of inducing a greater accumulation of total polyphenols and flavonoids without affecting the plant biomass. A high performance liquid chromatography (HPLC) analysis showed that the concentrations of many flavonoids significantly increased in response to UV-B treatment. The activities of three enzymes involved in the early steps of flavonoid biosynthesis, namely phenylalanine ammonia-lyase (PAL), cinnamate-4-hydroxylase (C4H), and 4-coumarate: coenzyme A (CoA) ligase (4CL), were significantly increased after the corresponding UV-B treatment. Compared with the control, the expression levels of several flavonoid biosynthesis genes (namely *BrPAL*, *BrC4H*, *Br4CL*, *BrCHS*, *BrF3H*, *BrF3′H*, *BrFLS*, *BrDFR*, *BrANS*, and *BrLDOX*) were also significantly up–regulated in the UV-B treatment group. The results suggest that appropriate preharvest UV-B supplementation could improve the nutritional quality of greenhouse-grown pakchoi by promoting the accumulation of antioxidants.

## 1. Introduction

In recent years, consumers have become more aware of the importance of dietary nutrition. High-quality functional foods, combining health and safety, are desired by consumers. Secondary plant metabolites (SPM), which include flavonoids, can not only be used as sunscreens by plant leaves to protect inner cells from harmful radiation, but are also considered to be the major bioactive compounds in edible plants with respect to human health benefits due to their potent antioxidant capacity [1,2]. *Brassica* species are known for their high contents of SPM, many of which are appreciated for their health-promoting effects. Kale (*Brassica* *oleracea* L.) has high concentrations of the flavonol aglycones kaempferol and quercetin, which show different antioxidant activities dependent on their chemical structure [3,4]. Several antioxidant phenolic compounds including flavonoids have been investigated and identified in Chinese cabbage (*Brassica rapa* L.) leaves [5]. Cabbage (*Brassica oleracea* L.) heads have important antioxidant and anti-inflammatory properties due to their rich glucosinolates content [6]. Pakchoi (*Brassica rapa* L.) is rich in SPM and contains numerous antioxidants, including flavonoids, hydroxycinnamic acids, carotenoids, chlorophylls, and glucosinolates [7,8]. With increasing attention being paid to the quality and safety of food, *Brassica* vegetables rich in antioxidants are gradually finding their way into our diets.

The biosynthesis of antioxidants in plant-derived food is regulated by many factors, including the light environment [3]. Ultraviolet-B (UV-B; 280–315 nm) radiation is an intrinsic part of the solar radiation that reaches the earth’s surface and plays an important role in regulating the growth, photosynthesis, and SPM of higher plants [9]. UV-B radiation resulted in changes in a number of antioxidants in different *Brassica* vegetables. Much evidence has indicated that the impact of UV-B radiation on plants depends upon the context, such as radiation dosage, exposure time, stress acclimation, nutritional status, and plant species [7,10,11]. Exposure to low doses of UV-B and UV during the late developmental stages of pakchoi resulted in higher concentrations of flavonoids, hydroxycinnamic acids, carotenoids, and chlorophylls [7]. Six leafy *Brassica* species were analyzed for their flavonoid glycoside accumulation after short-term UV-B treatment, which showed species-specific responses [12]. Blue light treatment after pre-exposure to UV-B stabilized the changes in flavonoid glycoside and led to a higher hydroxyl radical scavenging capacity in three different *Brassica* sprouts [4]. Moreover, the treatment of low, ecologically relevant UV-B levels did not result in adverse effects at the human cell level [13]. Cooking methods might affect the bioavailability and content of SPM. It was found that steaming retained more chlorophylls, glucosinolates, phenolic acids and flavonoid compounds than boiling in three different cultivars of pakchoi [8]. These findings suggested that the supplementation of white light with UV-B irradiation may be a sustainable tool for improving crop production quality and food safety. A crucial issue is the dosage of radiation necessary to optimize the biosynthesis of beneficial phytochemicals without affecting the times, quality, and quantity of the harvest.

Vegetables are the main source of antioxidants in the human diet and are essential in our daily lives. The consumption of diets high in vegetables has been associated with a lower risk of a number of chronic illnesses [14]. As a result of market demands and economic incentives, greenhouse vegetable production has been developed and rapidly expanded as an intensive form of agriculture, which provides consumers with sufficient vegetables in the on- and off-seasons in many developing countries [15]. However, most plastic films covering greenhouses or polytunnels almost completely absorb and hence block UV radiation (both UV-A and UV-B) reaching the plants, due mainly to the stabilizers used in the different materials to extend the longevity of the film [16]. Polycarbonate, polyethylene, and fiberglass are the most commonly used greenhouse covering materials, with the effect of excluding more than 90% of the incident UV-B radiation [17]. Therefore, most greenhouse-grown vegetables are basically protected from UV-B irradiation during the growth process, leading to a decrease in the content of antioxidants such as flavonoids. For example, the concentrations of flavonoid derivatives in the leaf blade of various pakchoi cultivars ranged from 15 to 39 mg·g^−1^ dry matter under field conditions, but only ranged from 4.7 to 16.7 mg·g^−1^ dry matter under greenhouse conditions. The concentrations of hydroxycinnamic acid derivatives were also significantly reduced [18,19]. So, it is of great significance to increase the accumulation of antioxidants in greenhouse vegetables by supplementation with UV-B.

Pakchoi is a leafy *Brassica* vegetable that is widely available in Asia and consumed in rising quantities in Europe with a high contents of antioxidants. Several studies have reported the effect of UV-B radiation on antioxidants in pakchoi as described earlier. However, to our knowledge, the direct correlation between antioxidants accumulation and antioxidant activity under different UV-B irradiation conditions has not yet been studied. The objective of this research is to identify the most appropriate UV-B treatment for improving the antioxidant capacity in pakchoi and to identify the antioxidants stimulated by UV-B radiation. The activities of the key enzymes and expression levels of the genes involved in flavonoid biosynthetic pathway were determined to explore the molecular mechanism of UV-B radiation in improving antioxidant capacity. Our results will provide a potential new tool by which to generate greenhouse vegetables enriched with antioxidants for either fresh consumption or as a source of functional foods.

## 2. Results and Discussion

### 2.1. Impacts of UV-B Radiation on Plant Growth and Biomass in Pakchoi

**Figure 1:**
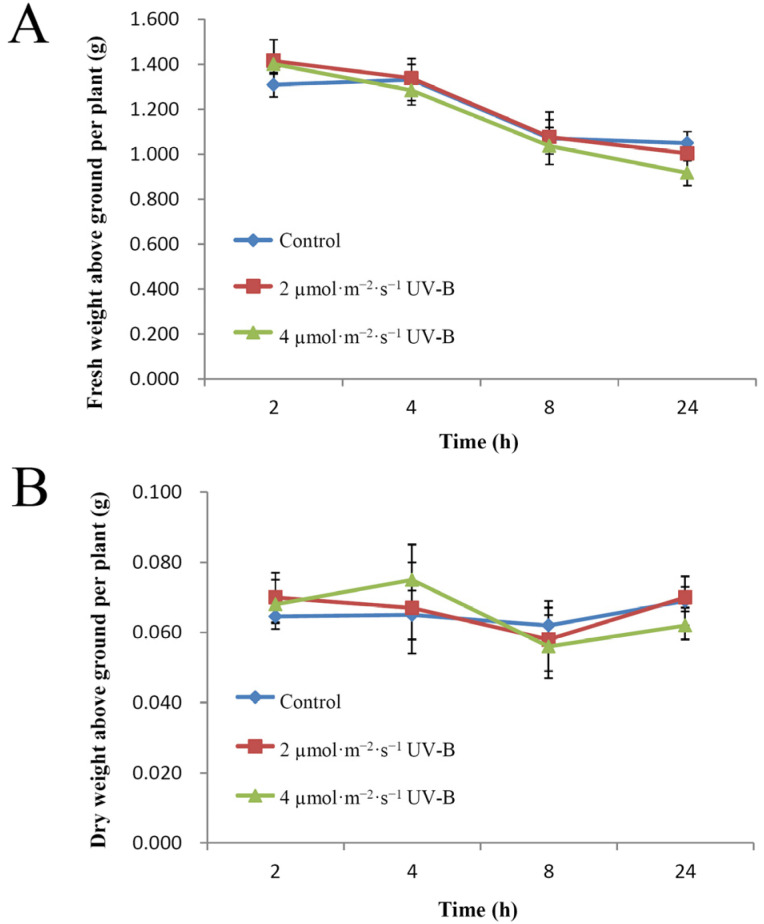
The effect of ultraviolet-B (UV-B) radiation on the above-ground biomass of pakchoi. (**A**) Fresh weight of 25-day-old seedlings treated with either 2 µmol·m^−2^·s^−1^ or 4 µmol·m^−2^·s^−1^ of UV-B irradiation for 2 h, 4 h, 8 h, or 24 h. (**B**) Dry weight of 25-day-old seedlings treated with either 2 µmol·m^−2^·s^−1^ or 4 µmol·m^−2^·s^−1^ of UV-B irradiation for 2 h, 4 h, 8 h, or 24 h. The plants without supplemental UV-B radiation served as controls. Data points are mean ± SE of three biological replicates. Significant differences between treated group and the control group at the same exposure period, identified by Student’s *t*-test analysis.

It is critical to identify the most appropriate UV-B radiation dosage and exposure period, which enhances antioxidant capacity without affecting the growth and morphology of pakchoi. We first assessed how pakchoi plant growth was impacted by two different doses (2 µmol·m^−2^·s^−1^ and 4 µmol·m^−2^·s^−1^) of UV-B radiation over each off our different exposure periods (2 h, 4 h, 8 h and 24 h). Compared with the control, there was no significant difference in fresh weight and dry weight under any UV-B radiation fluence rates (Figure 1). Our results are consistent with previous studies reporting that low, ecologically relevant UV-B levels do not affect plant growth [7,12]. As such, we further explored the impact of supplementary UV-B radiation on nutritional components and secondary metabolites in pakchoi.

### 2.2. UV-B Irradiation Effect on Total Antioxidant Capacity in Pakchoi

The total antioxidant capacity is often evaluated by 1,1–diphenyl–2–picrylhydrazyl (DPPH) scavenging activity, ferric reducing antioxidant power (FRAP), ABTS radical scavenging capacity, and oxygen radical absorption capacity assay in vegetables and fruits [20]. DPPH, as a stable free radical, has been widely employed to measure the radical scavenging effects of plant extracts [21]. The FRAP assay is a key method for assessing the total reduction capacity and offers a putative index of antioxidant capacity [22]. In the current study, the effect of UV-B radiation on total antioxidant capacity in pakchoi was assayed by measuring the DPPH scavenging activity and total reduction capacity.

**Figure 2:**
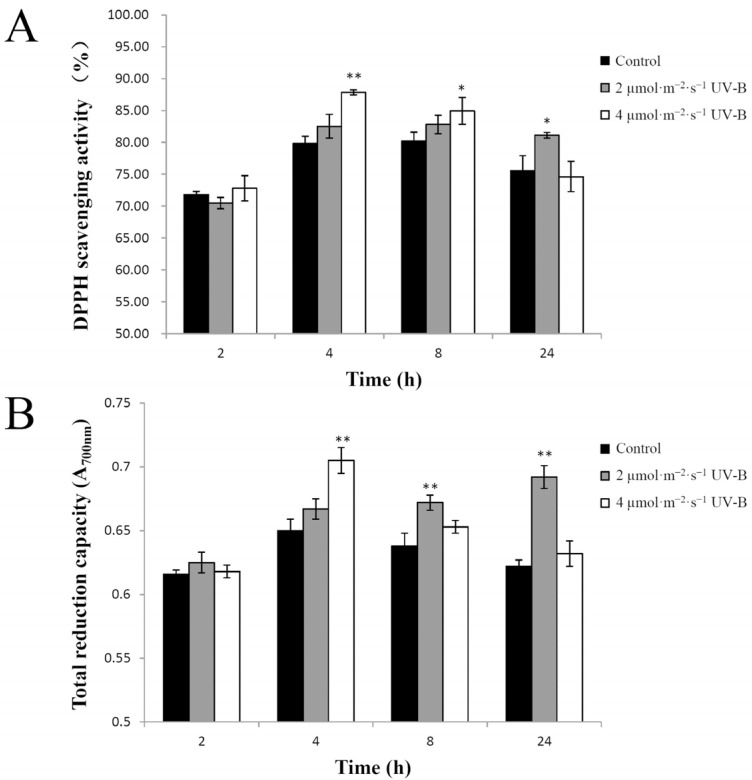
The effect of UV-B radiation on the total antioxidant capacity of pakchoi. (**A**) The 1,1–diphenyl-2-picrylhydrazyl (DPPH) scavenging activity of 25-day-old seedlings treated with either 2 µmol·m^−2^·s^−1^ or 4 µmol·m^−2^·s^−1^ of UV-B irradiation at 2 h, 4 h, 8 h, or 24 h. (**B**) The total reduction capacity of 25-day-old seedlings treated with either 2 µmol·m^−2^·s^−1^ or 4 µmol·m^−2^·s^−1^ of UV-B irradiation at 2 h, 4 h, 8 h, or 24 h. The plants not exposed to UV-B radiation served as controls. Three independent experiments were performed and data points represent the mean ± SE of three biological replicates. Asterisks indicate a significant difference (* *p* < 0.05; ** *p* < 0.01) to the corresponding control, using Student’s *t*-test.

There was no significant difference in DPPH scavenging activity between plants treated with either dose of UV-B radiation for 2 h and the control plants. Irradiation with 2 µmol·m^−2^·s^−1^ UV-B for 4 h or 8 h did not significantly increase the radical scavenging effects on DPPH. The DPPH scavenging activity of the plants irradiated with 2 µmol·m^−2^·s^−1^ UV-B for 24 h (81.12%) was significantly greater than that of the control (75.57%). The DPPH-scavenging activities of the plants irradiated with 4 µmol·m^−2^·s^−1^ UV-B for 4 h (87.84%; *p* < 0.01) or 8 h (84.95%; *p* < 0.05) were significantly greater than those of the controls (79.82% or 80.20%), although 4 µmol·m^−2^·s^−1^ UV-B irradiation for 24 h (74.64%) did not increase the scavenging effects on DPPH relative to the control (75.57%) (Figure 2A).

The effect of UV-B irradiation on total reduction capacity was similar to the effects on DPPH scavenging activity. The total reduction capacity of 2 µmol·m^−2^·s^−1^ UV-B radiation for either 8 h or 24 h and of 4 µmol·m^−2^·s^−1^ UV-B radiation for 4 h was significantly greater (*p* < 0.01) than that of the controls. No significant difference was found in the total reduction capacity between the plants exposed to other UV-B treatments and control plants (Figure 2B). These results indicated that the effect of UV-B radiation on total antioxidant capacity in pakchoi was dose-dependent, consistent with the previously reported results in the literature [7]. When the samples were collected immediately after the irradiation time-points, treatments with 2 µmol·m^−2^·s^−1^ UV-B radiation for 24 h or 4 µmol·m^−2^·s^−1^ UV-B radiation for 4 h had the greatest enhancement effect on antioxidant capacity in pakchoi. There was no significant stimulatory effect in response to 2 µmol·m^−2^·s^−1^ UV-B radiation for a shorter time or 4 µmol·m^−2^·s^−1^ UV-B radiation for a longer time. This effect may be related to different UV-B intensities activating particular signaling pathways, as described earlier [23]. However, the effect of UV-B radiation on antioxidant capacity varies over the collection time, which needs to be further studied.

**Figure 3:**
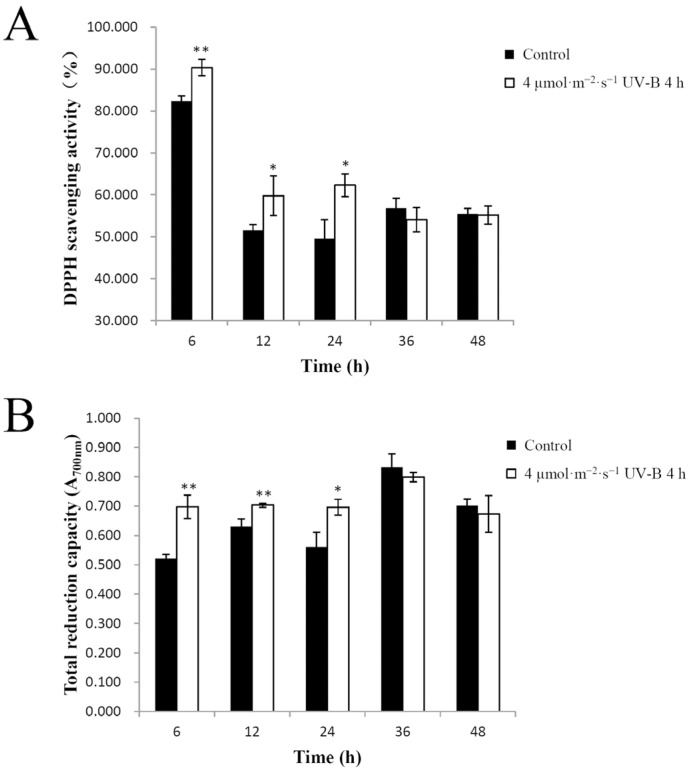
Duration of UV-B radiation effects on the antioxidant capacity of pakchoi. (**A**) The DPPH scavenging activity of 25-day-old seedlings at 6 h, 12 h, 24 h, 36 h, or 48 h after irradiation with 4 µmol·m^−2^·s^−1^ UV-B radiation for 4 h. (**B**) The total reduction capacity of 25-day-old seedlings at 6 h, 12 h, 24 h, 36 h, or 48 h after 4 µmol·m^−2^·s^−1^ UV-B radiation for 4 h. Plants grown without UV-B radiation served as controls. Three independent biological replicate experiments were performed; data points represent the mean ± SE of the three biological replicates. Asterisks indicate a significant difference (* *p* < 0.05; ** *p* < 0.01) relative to the corresponding control, using Student’s *t*-test.

In general, the effect of secondary metabolite accumulation induced by UV-B radiation lasts for some time. Su et al. reported that UV-B-induced anthocyanin accumulation in hypocotyls of radish sprouts could be sustained for a long time (more than 24 h) in the dark after irradiation [24]. To investigate whether a UV-B-induced increase of total antioxidant capacity continues after UV-B irradiation in pakchoi, the seedlings were first exposed to 4 µmol·m^−2^·s^−1^ UV-B for 4 h, and then transferred to darkness for 6, 12, 24, 36, and 48 h, respectively. The induction of DPPH scavenging activity could be maintained for 24 h in the dark following the radiation treatment (Figure 3A), although the enhancement effect was not apparent at 36 h or 48 h after treatment. As with DPPH scavenging activity, the induction of the total reduction capacity could also be maintained for 24 h in the dark after radiation (Figure 3B). Pakchoi is commonly consumed not only fresh (e.g., as salad), but also after cooking or fermentation. Furthermore, 21-day-old seedlings in three different cultivars of pakchoi were used to analyze the effect of domestic cooking methods (boiling and steaming) on secondary metabolites [8]. The production cycle of the pakchoi cultivar ‘Can Bai’ is 20–40 days, depending on growing temperature and consumer preference. The 25-day-old seedlings can be consumed for their high nutritional value, especially as baby salads. At the same time, there are some other ways to stabilize or further increase the enhancement effect of antioxidant capacity, such as blue light treatment after pre-exposure to UV-B as previously reported [4]. These might make the preharvest UV-B treatment an effective tool, allowing people to harvest more nutritious greenhouse-grown pakchoi in time. The decrease in DPPH scavenging activity from 6 h to 12 h might be due to the variable time of darkness in both the UV-B treatment and control groups. Dark treatments after UV-B irradiation can eliminate confounding factors such as incandescent and provide experimental evidence of energy efficiency and practical applications for enhancing the nutritional quality of pakchoi. The results showed that the energy could be saved for at least 6 h by the dark treatment.

### 2.3. Effects of UV-B Irradiation on Non-Enzymatic Antioxidants

Polyphenols, glutathione and ascorbate are considered to be potent non-enzymatic antioxidants in plants as they exhibit a high scavenging activity of harmful reactive oxygen species (ROS) [25,26,27]. Phenolic compounds are ubiquitous in the plant kingdom and constitute a large class of secondary metabolites, including phenolic acids, flavonoids, tannins, lignans, coumarins, and stilbenes [28]. Flavonoids are a biologically important group of phenolics, which have been recently suggested to contribute primary antioxidant functions in the responses of plants to a wide range of abiotic stresses, including UV-B radiation [1].

**Figure 4:**
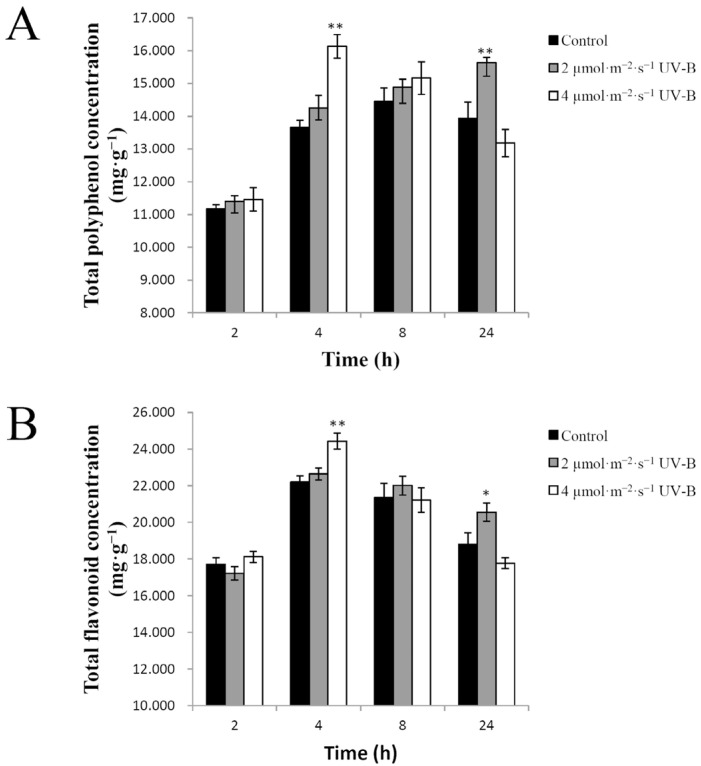
The effect of UV-B radiation on the antioxidant concentrations of pakchoi. (**A**) The total polyphenol concentration of 25-day-old seedlings treated with either 2 µmol·m^−2^·s^−1^ or 4 µmol·m^−2^·s^−1^ of UV-B radiation at 2 h, 4 h, 8 h, or 24 h. (**B**) The total flavonoid concentration of 25-day-old seedlings treated with either 2 µmol·m^−2^·s^−1^ or 4 µmol·m^−2^·s^−1^ of UV-B radiation at 2 h, 4 h, 8 h, or 24 h. The plants untreated with UV-B radiation served as controls. Three independent biological replicate experiments were performed; data points represent the mean ± SE of the three biological replicates. Asterisks indicate a significant difference (* *p* < 0.05; ** *p* < 0.01) relative to the corresponding control using Student’s *t*-test.

In the current study, the concentrations of total polyphenols, flavonoids, glutathione, and ascorbate in UV-B-treated and untreated pakchoi were determined and compared. As shown in Figure 4A, the concentration of total polyphenols increased very significantly after treatment with 2 µmol·m^−2^·s^−1^ UV-B radiation for 24 h or 4 µmol·m^−2^·s^−1^ UV-B radiation for 4 h compared with the control. The total polyphenol concentration increased from 13.94 mg·g^−1^ to 15.63 mg·g^−1^ (*p* <p0.01) after treatment with 2 µmol·m^−2^·s^−1^ UV-B radiation for 24 h or from 13.65 mg·g^−1^ to 16.13 mg·g^−1^ (*p* < 0.01) after treatment with 4 µmol·m^−2^·s^−1^ UV-B radiation for 4 h. No significant changes were observed in response to other UV-B irradiation treatments.

As the main phenolic compounds, the response of total flavonoid concentration to UV-B radiation was similar to that of total polyphenol concentration. The total flavonoid concentration increased significantly from 18.81 mg·g^−1^ to 20.57 mg·g^−1^ (*p* < 0.05) after treatment with 2 µmol·m^−2^·s^−1^ UV-B radiation for 24 h and from 22.20 mg·g^−1^ to 24.43 mg·g^−1^ (*p* < 0.01) after treatment with 4 µmol·m^−2^·s^−1^ UV-B radiation for 4 h. There was no significant increase in the total flavonoid concentration in response to other UV-B irradiated conditions compared with the control. The results showed that the induction of total polyphenols and flavonoids in UV-B-treated pakchoi was dependent on the radiation dosage and time (Figure 4B).

**Figure 5:**
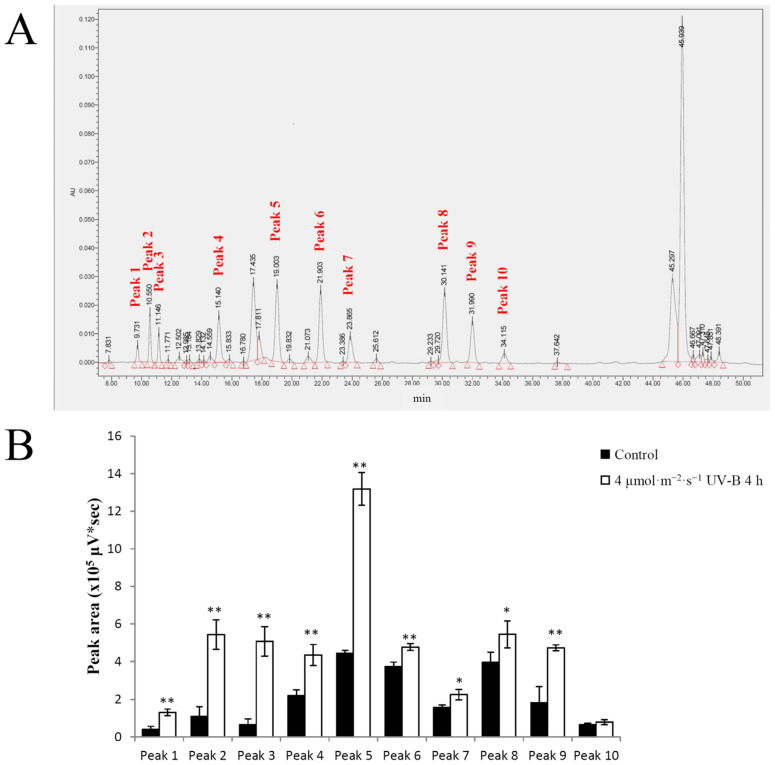
Determination of flavonoid concentrations in pakchoi in response to UV-B. (**A**) High performance liquid chromatography (HPLC) chromatogram of the flavonoids in extracts of 25-day-old seedlings. (**B**) The peak areas fractionated by HPLC. Twenty-five-day-old seedlings treated with 4 µmol·m^−2^·s^−1^ UV-B for 4 h were harvested for extraction. Twenty-five-day-old seedlings not treated with UV-B radiation served as the control. Three independent biological replicate experiments were performed; data points represent the mean ± SE of the three biological replicates. Asterisks indicate a significant difference (* *p* < 0.05; ** *p* < 0.01) relative to the corresponding control, using Student’s *t*-test.

There are many types of flavonoid, and changes in the concentrations of individual flavonoids in pakchoi between UV-B treatment and control groups were analyzed by high-performance liquid chromatography (HPLC). More than a dozen flavonoids were isolated from the pakchoi leaves based on their ultraviolet absorption spectrum and elution profile (Figure 5A). Among them, the peak areas of Peak 1, Peak 2, Peak 3, Peak 4, Peak 5, Peak 6 and Peak 9 increased very significantly (*p* < 0.01) in extracts of plants treated with 4 µmol·m^−2^·s^−1^ UV-B radiation for 4 h. The peak areas of Peak 7 and Peak 8 increased significantly (*p* < 0.05) in the extracts of plants treated with 4 µmol·m^−2^·s^−1^ UV-B radiation for 4 h. The peak area of Peak 10 did not change significantly (Figure 5B). In order to identify metabolic features, we carried out a liquid chromatography–mass spectrometry (LC–MS) analysis, and found that four of the peaks possibly representing flavonoids increased in response to UV-B (Figure S1). Due to the lack of suitable databases and standards, we could not confirm which specific type of flavonoids these peaks are. We speculate that they are most likely kaempferol glycosides according to the reported literature [8,11,12].

On the other hand, there was no obvious enhancement effect of UV-B radiation on glutathione and ascorbate concentrations in pakchoi (Figure S2). Pakchoi synthesizes comparatively high amounts of glucosinolates, most of which were not affected by reduced UV-B conditions during the late developmental stages of pakchoi [7]. Flavonoids were still not evaluated and further investigations on glucosinolates are required. Nonetheless, these results revealed that the increase in non-enzymatic antioxidant activity was mainly due to the accumulation of phenolic compounds, especially flavonoids. This finding is in agreement with previous studies that showed that UV-B radiation can induce the biosynthesis of flavonoids in a range of plants [29,30,31,32].

### 2.4. Effects of UV-B Radiation on Flavonoid Biosynthesis Enzymes

The phenolic and flavonoid compound biosynthesis pathway is one of the most extensively studied areas of SPM. Flavonoids are synthesized via the shikimate-phenylpropanoid-flavonoid pathways in plants as documented in recent literature [33,34]. The phenylpropanoid pathway begins from the aromatic amino acids phenylalanine and tyrosine, which are synthesized by the shikimate pathway, to generate 4-coumaroyl-CoA, which is utilized in the flavonoid pathway. A number of important enzymes are involved in this process, such as PAL, C4H, 4CL, CHS, F3H, F3′H, FLS, DFR, and ANS. Among them, PAL, C4H and 4CL are three major enzymes in the phenylpropanoid pathway.

**Figure 6:**
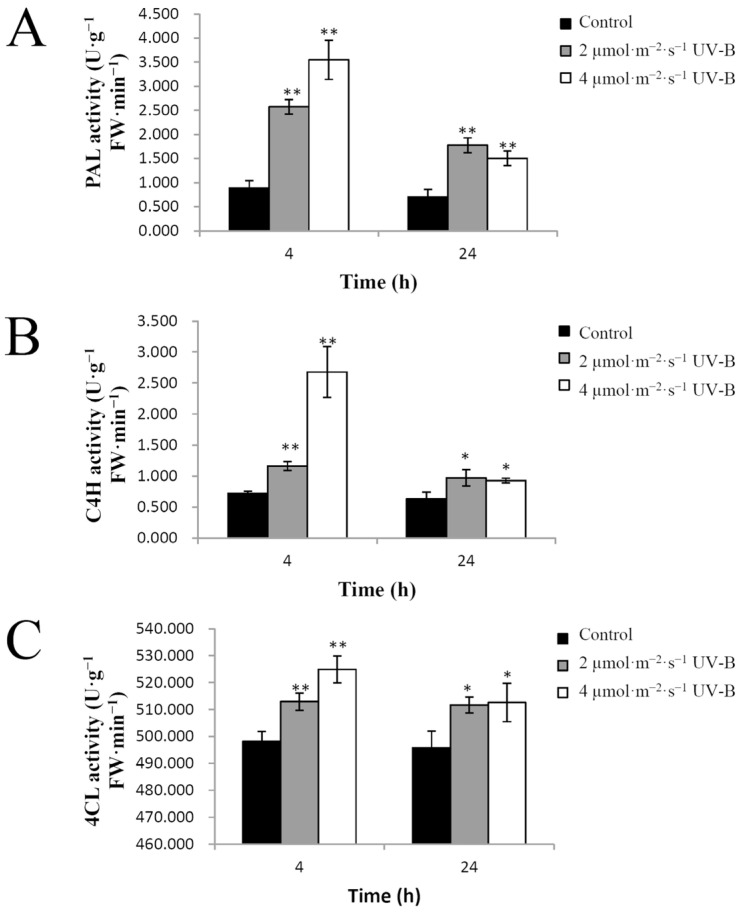
The effect of UV-B radiation on the activities of flavonoid biosynthesis enzymes in pakchoi. (**A**) The activity of PAL in 25-day-old seedlings treated with either 2 µmol·m^−2^·s^−1^ or 4 µmol·m^−2^·s^−1^ of UV-B radiation at 4 h or 24 h. (**B**) The activity of C4H in 25-day-old seedlings treated with either 2 µmol·m^−2^·s^−1^ or 4 µmol·m^−2^·s^−1^ of UV-B radiation at 4 h or 24 h. (**C**) The activity of 4CL in 25-day-old seedlings treated with either 2 µmol·m^−2^·s^−1^ or 4 µmol·m^−2^·s^−1^ of UV-B radiation at 4 h or 24 h. The plants without UV-B radiation served as controls. Three independent biological replicate experiments were performed; data points represent the mean ± SE of three biological replicates. Asterisks indicate a significant difference (* *p* < 0.05; ** *p* < 0.01) relative to the corresponding control, using Student’s *t*-test.

The effects of UV-B radiation on PAL, C4H and 4CL activities in pakchoi were examined in this study. PAL activity increased very significantly (*p* < 0.01) in response to 2 µmol·m^−2^·s^−1^ or 4 µmol·m^−2^·s^−1^ UV-B radiation for 4 h or 24 h compared with the control (Figure 6A). The activity of C4H increased very significantly (*p* < 0.01) after exposure to 2 µmol·m^−2^·s^−1^ or 4 µmol·m^−2^·s^−1^ UV-B radiation for 4 h compared with the control, whereas the increase after 24 h was only significant (*p* < 0.05) (Figure 6B). The activity of 4CL increased significantly (*p* < 0.05) in response to 2 µmol·m^−2^·s^−1^ or 4 µmol·m^−2^·s^−1^ UV-B radiation for 24 h compared with the control and very significantly (*p* < 0.01) after 2 µmol·m^−2^·s^−1^ or 4 µmol·m^−2^·s^−1^ UV-B radiation for 4 h compared with the control (Figure 6C). The results revealed that the observed stimulatory effect of UV-B radiation on the production of flavonoids could be explained by the induction of the activities of important enzymes in the flavonoid biosynthesis pathway, a finding which was consistent with previous reports from other plant species [24,26,35].

### 2.5. UV-B Effect on the Expression of Flavonoid Biosynthesis Genes

**Figure 7:**
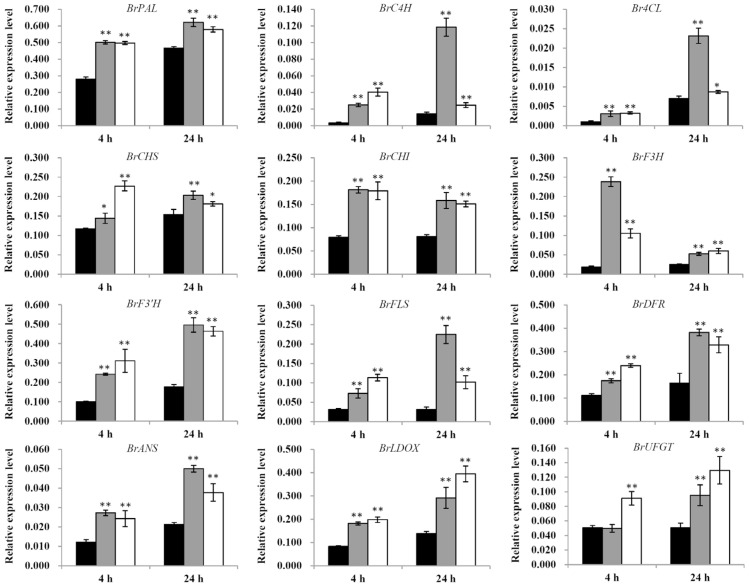
Relative expression levels of the genes related to flavonoid biosynthesis in response to UV-B radiation. Black columns represent control; gray columns represent 2 µmol·m^−2^·s^−1^ UV-B; white columns represent 4 µmol·m^−2^·s^−1^ UV-B. Gene expression values are relative to reference *BrActin2* expression; data points represent the mean ± SE of three biological replicates. Asterisks indicate a significant difference (* *p* < 0.05; ** *p* < 0.01) relative to the corresponding control using Student’s *t*-test.

Anthocyanins and flavonols are the two major classes of flavonoid compounds, in terms of their role in protecting plants against abiotic and biotic stresses. A total of 73 anthocyanin biosynthetic genes in *Brassica rapa* have been identified using comparative genomic analyses between *Brassica rapa* and *Arabidopsis thaliana* [36]. The expression levels of some of these flavonoid biosynthesis genes in response to 2 or 4 µmol·m^−2^·s^−1^ UV-B irradiation for 4 h or 24 h were analyzed in pakchoi in the current study. The expression of each of the three major genes of the phenylpropanoid pathway, *BrPAL, BrC4H*, and *Br4CL*, was upregulated significantly (*p* < 0.05 or *p* < 0.01) in each of the UV-B treatment groups compared with the controls, a finding which was basically consistent with the results of the corresponding enzyme activity analysis. The expression of the early biosynthesis genes in the flavonoid pathway, *BrCHS*, *BrCHI*, *BrF3H*, *BrF3′H*, and *BrFLS*, and of the late biosynthesis genes in the flavonoid pathway, *BrDFR*, *BrANS*, *BrLDOX*, and *BrUFGT*, were upregulated significantly (*p* < 0.05) or very significantly (*p* < 0.01) after UV-B irradiation, compared with the control. Overall, the highest expression level occurred at 2 µmol·m^−2^·s^−1^ UV-B radiation for 24 h and 4 µmol·m^−2^·s^−1^ UV-B radiation for 4 h, findings which were consistent with the previous enzyme activity results (Figure 7). These results showed that the irradiation-induced increases in concentrations of flavonoids were associated with corresponding increases in the expression of flavonoid biosynthesis genes.

All of the above results indicated that the changes in gene expression, enzyme activity, and antioxidant concentration in response to supplementary UV-B radiation are basically consistent, as previously reported [37]. Moreover, the gene expression levels and biosynthetic enzyme activities are more sensitive to UV-B radiation than the flavonoid antioxidant concentration levels.

## 3. Materials and Methods

### 3.1. Plant Materials and Growth Conditions

A local commercial cultivar of pakchoi, ‘Can Bai’ (by Zhejiang Academy of Agricultural Sciences, Hangzhou, China), was used in the experiments. The plants were cultivated in an illuminated growth chamber (26 °C, 12 h/12 h light/dark cycle regime) in soil (peat, pH 5.5–6.5; Fafard, Saint-Bonaventure, QC, Canada). Water was supplied as required by the plants and fertilizer was administered weekly with Hoagland’s nutrient solution. The light intensity of incandescent was 100 µmol·m^−2^·s^−1^ (400–700 nm). For experimentation, 25-day-old seedlings were supplemented with (treatment group) or without (control group) UV-B irradiation and tissue was collected immediately after the irradiation time-points for analysis. Fifteen plants were pooled for one biological replicate, and all experiments were performed in triplicate.

### 3.2. Radiation Procedure

Pakchoi seedlings were placed on shelves and exposed to the supplementary UV-B radiation, at doses of 2 µmol·m^−2^·s^−1^ (equals 0.7 W·m^−2^) or 4 µmol·m^−2^·s^−1^ (equals 1.4 W·m^−2^) for 2 h, 4 h, 8 h, or 24 h. UV-B radiation was supplied by five fluorescent lamps (40 W 12RS, Beijing Lighting Research Institute, Beijing, China), whereby the UV-B emission peaked at 313 nm. The desired radiation dose was obtained by changing the number of UV-B lamps and the distance between the lamps and the plants. UV-B radiation was measured using an Optronics Model 720 spectroradiometer (Beijing Normal University Optronics Factory, Beijing, China), with a spectral range of 280 to 400 nm.

### 3.3. DPPH Scavenging Assay

The DPPH scavenging activity assay was performed according to the method reported by Alhaithloul et al. [38]. Aliquots (0.2 g) of the dried samples were extracted with 45 mL 70% methanol in a water bath at 70 °C for 60 min and centrifuged for 15 min at 4700× *g*. The supernatant was retained and used to determine DPPH scavenging activity. An aliquot (4 mL) of 2.0 × 10^−4^ mmol·L^−1^ DPPH solution in 70% ethanol was added to 1 mL of the supernatant. The mixture was allowed to incubate for 30 min at room temperature in the dark, after which the absorbance at 517 nm was measured.

### 3.4. Determination of Total Reduction Capacity

The FRAP assay was performed to determine the total reduction capacity according to the procedure reported previously [6]. Aliquots (0.2 g) of the dried samples were extracted with 45 mL 70% methanol in a water bath at 70°C for 60 min and centrifuged for 15 min at 4700× *g*. The supernatant was retained and used for assays. The FRAP reagent included a 300 mM acetate buffer (pH 3.6), 10 mM 2,4,6-tris(2-pyridyl)-1,3,5-triazine in 40 mM HCl, and 20 mM FeCl_3_ in the ratio 10:1:1 (*v*:*v*:*v*). An aliquot (3 mL) of the FRAP reagent was mixed with 100 μL of the sample extract in a test tube, vortexed and incubated at 37 °C for 30 min in a water bath. The absorbance was measured at 700 nm.

The total phenolic concentration was measured using the Folin–Ciocalteu method as described previously with some modifications [39]. In brief, 0.5 mL of sample extract was mixed with 1.8 mL of 0.1 N Folin–Ciocalteu reagent (Sangon Biotech, Shanghai, China). After incubating for 5 min at room temperature, the reaction was stopped by the addition of 1.2 mL of an aqueous solution of 7.5% sodium carbonate. Then, the absorbance was measured at 765 nm. Gallic acid was used as the standard for a calibration curve, and the results were expressed as gallic acid equivalents.

The determination of total flavonoid concentration was performed as described previously with slight modifications [40]. Then, 2 mL of the sample extract was placed in a 10-mL volumetric flask and 0.5 mL of 5% NaNO_2_ was added, following which, 0.5 mL of 10% AlCl_3_ was added. After 6 min, 4 mL of 4% NaOH was added, and the total volume was 10 mL, with 70% ethanol. The solution was mixed well again, and the absorbance was measured at 510 nm. Rutin was used as the standard for a calibration curve, and the results were expressed as rutin equivalents.

### 3.5. Analysis of Flavonoids by HPLC and LC–MS

The HPLC and LC–MS analyses of flavonoids were carried out as described before with some modifications [41]. A tissue sample (0.1 g dry weight) was extracted in 1.5 mL of 80% methanol in the water bath for 60 min at 70 °C, centrifuged for 15 min at 4700× *g* and then filtered through a 0.22-µm pore size filter (Millipore, Billerica, MA, USA) prior to analysis. An HPLC analysis was performed on a Waters 2695 Alliance HPLC system (Waters, Milford, MA, USA) equipped with a photodiode array detector. A C18 column (4.6 mm i.d. × 250 mm) (Waters, Milford, MA, USA) was used with a flow rate of 1 mL·min^−1^ at 25 °C. Gradient elution was employed using mobile phases of 0.1% trifluoroacetic acid (A) and acetonitrile (B) (Supplementary Table S1). Spectra were measured at a wavelength of 350 nm, and individual flavonoids were identified by comparing the retention time and UV spectra. LC–MS analyses were carried out using an LCQ ion trap mass spectrometer (Finnigan MAT, San Jose, CA, USA) equipped with an ESI source in the positive ion mode. Helium was used as the buffer gas and nitrogen was used as the dry gas (12 L·min^−1^, 350 °C). The heated metal capillary temperature was 180 °C. The electrospray voltage was at 4.5 kV. The data were analyzed using a DataAnalysis Compass.

### 3.6. Assay of Flavonoid Biosynthesis Enzyme Activities

PAL activity was determined as described previously with some modifications [42]. Briefly, fresh samples (0.5 g) were homogenized in 10 mL of pre–cooled extractant solution (0.01 mol·L^−1^ boric acid buffer, pH 8.8; containing 5 mmol·L^−1^ β–mercaptoethanol) and centrifuged for 20 min at 4 °C at 9600× *g*. The supernatant was retained and used to determine the PAL activity. The reaction mixture consisted of 100 μL supernatant, 3.0 mL of 0.01 mol·L^−1^ sodium borate (pH 8.8; containing 0.005 mol·L^−1^ β–mercaptoethanol) and 700 μL of 0.01 mol·L^−1^ L–phenylalanine. The reaction mixture was incubated at 30 °C for 30 min and the reaction was stopped by adding 0.2 mL of 6 mol·L^−1^ HCl. PAL activity was spectrophotometrically measured by monitoring the absorbance at 290 nm. Then, C4H and 4CL activity were determined according to the procedures reported previously [43,44].

### 3.7. RNA Extraction and Quantitative Reverse Transcription PCR (qRT–PCR) Analysis

Total RNA was isolated with the OmniPlant RNA Kit (DNase I) CW2598 (CWBIO, Beijing, China) as previously described [45]. HiFiScript gDNA Removal cDNA Synthesis Kit CW2582 (CWBIO, Beijing, China) was used to achieve first-strand cDNA synthesis from approximately 1 µg of total RNA. qRT–PCR was performed using the iQ SYBR Green Supermix (Bio–Rad, Hercules, CA, USA) and run on the ABI Prism 7000 system (Applied Biosystems, Foster City, CA, USA). The sequences of the genes studied in this article (*BrActin*, Bra022356; *BrPAL*, Bra005221; *BrC4H*, Bra018311; *Br4CL*, Bra030429; *BrCHS*, Bra008792; *BrCHI*, Bra007142; *BrF3H*, Bra036828; *BrF3’H*, Bra009312; *BrFLS*, Bra009358; *BrDFR*, Bra027457; *BrANS*, Bra013652; *BrLDOX*, Bra019350; *BrUFGT*, Bra023954) were derived from a previously published paper [36] and *Brassica* database BRAD (http://brassicadb.cn, accessed on 20 January 2022) [46]. Furthermore, *BrActin2* was used as the reference housekeeping gene. The relative expression level of the target genes was normalized against the reference housekeeping gene [47]. The primers used in the qRT–PCR are listed in Supplementary Table S2.

## 4. Conclusions

Pakchoi is a very popular vegetable, rich in antioxidants with health benefits for consumers. However, greenhouse cultivation negatively affects the biosynthesis of antioxidants in pakchoi by interfering with incident UV-B. Preharvest UV-B supplementation has proved to be a very effective measure by which to improve the nutritional quality of pakchoi by promoting the accumulation of antioxidants in greenhouse-grown plants. Since the effects of UV-B radiation on plants depend on the radiation dose, exposure time, and plant species, we evaluated the effects of two different doses of UV-B radiation on pakchoi for four different irradiation periods. Our results showed that the appropriate UV-B irradiation treatments (4 µmol·m^−2^·s^−1^ for 4 h or 2 µmol·m^−2^·s^−1^ for 24 h) could significantly upregulate the expression of flavonoid biosynthesis genes (*BrPAL*, *BrC4H*, *Br4CL*, *BrCHS*, *BrF3H*, *BrF3′H*, *BrFLS*, *BrDFR*, *BrANS,* and *BrLDOX*), increase the activities of the most important enzymes (PAL, C4H and 4CL), promote the accumulation of flavonoids, and eventually lead to the improvement of antioxidant activity in pakchoi. This study provides a basis for future comprehensive studies on the metabolic mechanism of flavonoid biosynthesis, and new insight into an enhancement of the nutritional quality of greenhouse-grown vegetables.

## Data Availability

Not applicable.

## Supplementary Materials

The following supporting information can be downloaded at: https://www.mdpi.com/article/10.3390/plants11060766/s1, Figure S1. Mass spectrometric analysis of the flavonoids which increased in response to 4 µmol·m^−2^·s^−1^ UV-B radiation for 4 h. Figure S2: The effect of UV-B radiation on the antioxidant concentrations of pakchoi. (**A**) The glutathione concentration of 25-day-old seedlings treated with either dosage (2 µmol·m^−2^·s^−1^ or 4 µmol·m^−2^·s^−1^) of UV-B radiation at 4 h or 24 h. (**B**) The ascorbate concentration of 25-day-old seedlings treated with either dosage (2 µmol·m^−2^·s^−1^ or 4 µmol·m^−2^·s^−1^) of UV-B radiation at 4 h or 24 h. The plants without UV-B radiation served as controls. Three biologically independent replicate experiments were performed; data points represent the mean ± SE of three biological replicates. Asterisks indicate a significant difference (* *p* < 0.05; ** *p* < 0.01) relative to the corresponding control, using Student’s *t*-test; Table S1: Gradient elution program for HPLC analysis; Table S2: Primers used in quantitative reverse transcription PCR (qRT–PCR).

## References

[1] Brunetti C., Di Ferdinando M., Fini A., Pollastri S., Tattini M. (2013). Flavonoids as antioxidants and developmental regulators: Relative significance in plants and humans. Int. J. Mol. Sci..

[2] Del Rio D., Rodriguez-Mateos A., Spencer J.P., Tognolini M., Borges G., Crozier A. (2013). Dietary (poly)phenolics in human health: Structures, bioavailability, and evidence of protective effects against chronic diseases. Antioxid. Redox Signal..

[3] Neugart S., Klaring H.P., Zietz M., Schreiner M., Rohn S., Kroh L.W., Krumbein A. (2012). The effect of temperature and radiation on flavonol aglycones and flavonol glycosides of kale (*Brassica oleracea* var. sabellica). Food Chem..

[4] Neugart S., Majer P., Schreiner M., Hideg É. (2021). Blue light treatment but not green light treatment after pre-exposure to UV-B stabilizes flavonoid glycoside changes and corresponding biological effects in three different Brassicaceae sprouts. Front. Plant Sci..

[5] Seong G.U., Hwang I.W., Chung S.K. (2016). Antioxidant capacities and polyphenolics of Chinese cabbage (*Brassica rapa* L. ssp. Pekinensis) leaves. Food Chem..

[6] Rokayya S., Li C.J., Zhao Y., Li Y., Sun C.H. (2014). Cabbage (*Brassica oleracea* L. var. *capitata*) phytochemicals with antioxidant and anti-inflammatory potential. Asian Pac. J. Cancer Prev..

[7] Heinze M., Hanschen F.S., Wiesner-Reinhold M., Baldermann S., Gräfe J., Schreiner M., Neugart S. (2018). Effects of developmental stages and reduced UVB and low UV conditions on plant secondary metabolite profiles in pak choi (*Brassica rapa* subsp. *chinensis*). J. Agric. Food Chem..

[8] Chen X.M., Hanschen F.S., Neugart S., Schreiner M., Vargas S.A., Gutschmann B., Baldermann S. (2019). Boiling and steaming induced changes in secondary metabolites in three different cultivars of pak choi (*Brassica rapa* subsp. *chinensis*). J. Food Compos. Anal..

[9] Jansen M.A., Bornman J.F. (2012). UV-B radiation: From generic stressor to specific regulator. Physiol. Plant.

[10] Reifenrath K., Muller C. (2007). Species-specific and leaf-age dependent effects of ultraviolet radiation on two Brassicaceae. Phytochemistry.

[11] Harbaum-Piayda B., Walter B., Bengtsson G.B., Hubbermann E.M., Bilger W., Schwarz K. (2010). Influence of pre-harvest UV-B irradiation and normal or controlled atmosphere storage on flavonoid and hydroxycinnamic acid contents of pak choi (*Brassica campestris* L. ssp *chinensis* var. communis). Postharvest Biol. Tech..

[12] Neugart S., Bumke-Vogt C. (2021). Flavonoid glycosides in *Brassica* species respond to UV-B depending on exposure time and adaptation time. Molecules.

[13] Wiesner-Reinhold M., Gomas J.V.D., Herz C., Tran H.T.T., Baldermann S., Neugart S., Filler T., Glaab J., Einfeldt S., Schreiner M. (2021). Subsequent treatment of leafy vegetables with low doses of UVB-radiation does not provoke cytotoxicity, genotoxicity, or oxidative stress in a human liver cell model. Food Biosci..

[14] Hung H.C., Joshipura K.J., Jiang R., Hu F.B., Hunter D., Smith-Warner S.A., Colditz G.A., Rosner B., Spiegelman D., Willett W.C. (2004). Fruit and vegetable intake and risk of major chronic disease. J. Natl. Cancer Inst..

[15] Yang L.Q., Huang B.A., Hu W.Y., Chen Y., Mao M.C., Yao L.P. (2014). The impact of greenhouse vegetable farming duration and soil types on phytoavailability of heavy metals and their health risk in eastern China. Chemosphere.

[16] Mormile P., Rippa M., Graziani G., Ritieni A. (2019). Use of greenhouse-covering films with tailored UV-B transmission dose for growing ‘medicines’ through plants: Rocket salad case. J. Sci. Food Agric..

[17] Yoshida H., Nishikawa T., Hikosaka S., Goto E. (2021). Effects of nocturnal UV-B irradiation on growth, flowering, and phytochemical concentration in leaves of greenhouse-grown red perilla. Plants.

[18] Harbaum B., Hubbermann E.M., Wolff C., Herges R., Zhu Z., Schwarz K. (2007). Identification of flavonoids and hydroxycinnamic acids in pak choi varieties (*Brassica campestris* L. ssp. *chinensis* var. *communis*) by HPLC-ESI-MSn and NMR and their quantification by HPLC-DAD. J. Agric. Food Chem..

[19] Harbaum B., Hubbermann E.M., Zhu Z., Schwarz K. (2008). Free and bound phenolic compounds in leaves of pak choi (*Brassica campestris* L. ssp. *chinensis* var. *communis*) and Chinese leaf mustard (*Brassica juncea* Coss). Food chem..

[20] Dudonne S., Vitrac X., Coutiere P., Woillez M., Merillon J.M. (2009). Comparative study of antioxidant properties and total phenolic content of 30 plant extracts of industrial interest using DPPH, ABTS, FRAP, SOD, and ORAC assays. J. Agric. Food Chem..

[21] Nenadis N., Tsimidou M. (2002). Observations on the estimation of scavenging activity of phenolic compounds using rapid 1,1-diphenyl-2-picrylhydrazyl (DPPH•) tests. J. Am. Oil Chem. Soc..

[22] Benzie I.F.F., Strain J.J. (1996). The ferric reducing ability of plasma (FRAP) as a measure of “antioxidant power”: The FRAP assay. Anal. Biochem..

[23] Brown B.A., Jenkins G.I. (2008). UV-B signaling pathways with different fluence-rate response profiles are distinguished in mature Arabidopsis leaf tissue by requirement for UVR8, HY5, and HYH. Plant Physiol..

[24] Su N.N., Lu Y.W., Wu Q., Liu Y.Y., Xia Y., Xia K., Cui J. (2016). UV-B-induced anthocyanin accumulation in hypocotyls of radish sprouts continues in the dark after irradiation. J. Sci. Food Agric..

[25] Usano-Alemany J., Panjai L. (2015). Effects of increasing doses of UV-B on main phenolic acids content, antioxidant activity and estimated biomass in lavandin (*Lavandula x intermedia*). Nat. Prod. Commun..

[26] Takshak S., Agrawal S.B. (2015). Defence strategies adopted by the medicinal plant *Coleus forskohlii* against supplemental ultraviolet-B radiation: Augmentation of secondary metabolites and antioxidants. Plant Physiol. Biochem..

[27] Singh V.P., Srivastava P.K., Prasad S.M. (2012). Differential effect of UV-B radiation on growth, oxidative stress and ascorbate-glutathione cycle in two cyanobacteria under copper toxicity. Plant Physiol. Biochem..

[28] Dai J., Mumper R.J. (2010). Plant phenolics: Extraction, analysis and their antioxidant and anticancer properties. Molecules.

[29] Nascimento L.B., Leal-Costa M.V., Menezes E.A., Lopes V.R., Muzitano M.F., Costa S.S., Tavares E.S. (2015). Ultraviolet-B radiation effects on phenolic profile and flavonoid content of *Kalanchoe pinnata*. J. Photochem. Photobiol. B..

[30] Sun M.Y., Gu X.D., Fu H.W., Zhang L., Chen R.Z., Cui L., Zheng L.H., Zhang D.W., Tian J.K. (2010). Change of secondary metabolites in leaves of *Ginkgo biloba* L. in response to UV-B induction. Innov. Food Sci. Emerg. Technol..

[31] Martinez-Luscher J., Torres N., Hilbert G., Richard T., Sanchez-Diaz M., Delrot S., Aguirreolea J., Pascual I., Gomes E. (2014). Ultraviolet-B radiation modifies the quantitative and qualitative profile of flavonoids and amino acids in grape berries. Phytochemistry.

[32] Avena-Bustillos R.J., Du W.X., Woods R., Olson D., Breksa A.P., McHugh T.H. (2012). Ultraviolet-B light treatment increases antioxidant capacity of carrot products. J. Sci. Food Agric..

[33] Saito K., Yonekura-Sakakibara K., Nakabayashi R., Higashi Y., Yamazaki M., Tohge T., Fernie A.R. (2013). The flavonoid biosynthetic pathway in Arabidopsis: Structural and genetic diversity. Plant Physiol. Biochem..

[34] Winkel-Shirley B. (2001). Flavonoid biosynthesis. A colorful model for genetics, biochemistry, cell biology, and biotechnology. Plant Physiol..

[35] Jiao J., Gai Q.Y., Wang W., Luo M., Gu C.B., Fu Y.J., Ma W. (2015). Ultraviolet radiation-elicited enhancement of isoflavonoid accumulation, biosynthetic gene expression, and antioxidant activity in *Astragalus membranaceus* hairy root cultures. J. Agric. Food Chem..

[36] Guo N., Cheng F., Wu J., Liu B., Zheng S.N., Liang J.L., Wang X.W. (2014). Anthocyanin biosynthetic genes in *Brassica rapa*. BMC genom..

[37] Nguyen C.T.T., Lim S., Lee J.G., Lee E.J. (2017). VcBBX, VcMYB21, and VcR2R3MYB transcription factors are involved in UV–B-induced anthocyanin biosynthesis in the peel of harvested blueberry fruit. J. Agric. Food Chem..

[38] Alhaithloul H.A., Soliman M.H., Ameta K.L., El-Esawi M.A., Elkelish A. (2020). Changes in ecophysiology, osmolytes, and secondary metabolites of the medicinal plants of *Mentha piperita* and *Catharanthus roseus* subjected to drought and heat stress. Biomolecules.

[39] Loayza F.E., Brecht J.K., Simonne A.H., Plotto A., Baldwin E.A., Bai J., Lon-Kan E. (2021). A brief hot-water treatment alleviates chilling injury symptoms in fresh tomatoes. J. Sci. Food Agric..

[40] Jia Z.S., Tang M.C., Wu J.M. (1999). The determination of flavonoid contents in mulberry and their scavenging effects on superoxide radicals. Food Chem..

[41] Ni J., Hao J., Jiang Z.F., Zhan X.R., Dong L.X., Yang X.L., Sun Z.H., Xu W.Y., Wang Z.K., Xu M.J. (2017). NaCl induces flavonoid biosynthesis through a putative novel pathway in post-harvest Ginkgo leaves. Front. Plant Sci..

[42] Huang J., Gu M., Lai Z., Fan B., Shi K., Zhou Y.H., Yu J.Q., Chen Z. (2010). Functional analysis of the Arabidopsis *PAL* gene family in plant growth, development, and response to environmental stress. Plant Physiol..

[43] Lamb C.J., Rubery P.H. (1975). A spectrophotometric assay for trans-cinnamic acid 4-hydroxylase activity. Anal. Biochem..

[44] Knobloch K.H., Hahlbrock K. (1975). Isoenzymes of p-coumarate: CoA ligase from cell suspension cultures of *Glycine max*. Eur. J. Biochem..

[45] Hao J., Lou P.P., Han Y.D., Chen Z.H., Chen J.M., Ni J., Yang Y.J., Jiang Z.F., Xu M.J. (2021). GrTCP11, a cotton TCP transcription factor, inhibits root hair elongation by down-regulating jasmonic acid pathway in *Arabidopsis thaliana*. Front. Plant Sci..

[46] Cheng F., Liu S.Y., Wu J., Fang L., Sun S.L., Liu B., Li P.X., Hua W., Wang X.W. (2011). BRAD, the genetics and genomics database for Brassica plants. BMC Plant Biol..

[47] Long L., Yang W.W., Liao P., Guo Y.W., Kumar A., Gao W. (2019). Transcriptome analysis reveals differentially expressed ERF transcription factors associated with salt response in cotton. Plant Sci..

