# Supplementary material for: Ultraviolet-B Irradiation Increases Antioxidant Capacity of Pakchoi (Brassica rapa L.) by Inducing Flavonoid Biosynthesis
Source: Plants (Basel). 2022 Mar 13;11(6):766. doi: 10.3390/plants11060766 (PMC8949486; doi:10.3390/plants11060766)
Supplement: Supplementary file 1 [file plants-11-00766-s001.zip › plants-1598191-supplementary.pdf]

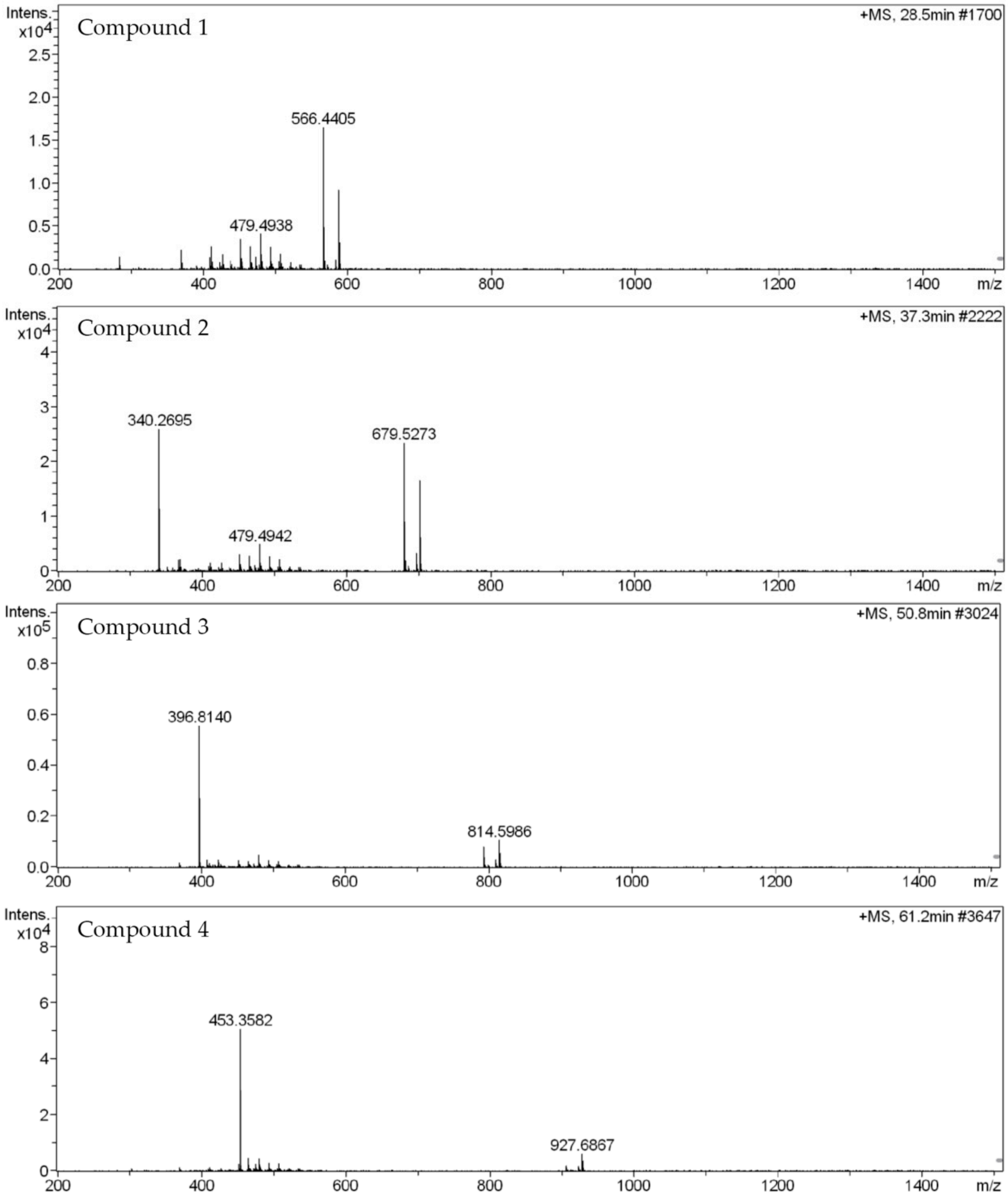

Figure S1. Mass spectrometric analysis of the flavonoids which increased in response to 4 μmol·m^{-2}·s^{-1} UV-B radiation for 4 h.

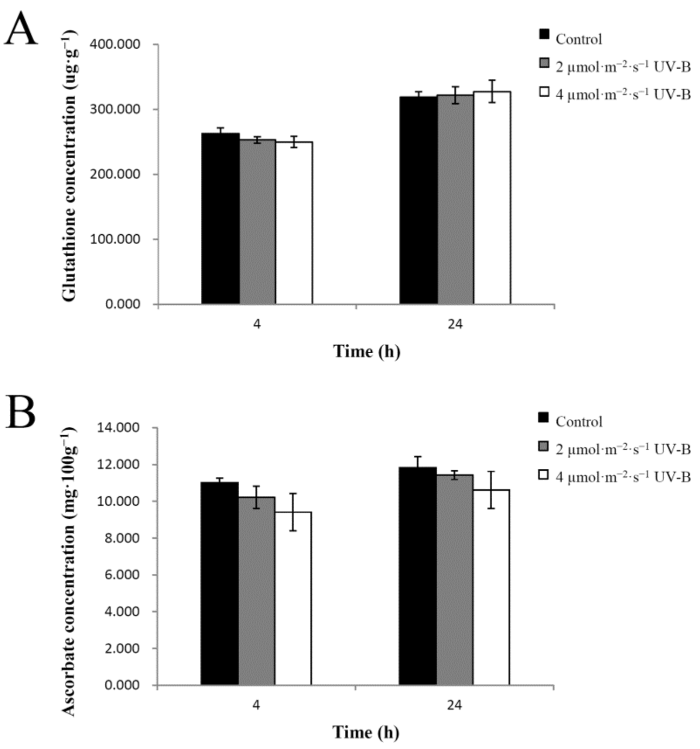

Figure S2. The effect of UV-B radiation on the antioxidant concentrations of pakchoi. (**A**) The glutathione concentration of 25-day-old seedlings treated with either dosage (2 μmol·m^{-2}·s^{-1} or 4 μmol·m^{-2}·s^{-1}) of UV-B radiation at 4 h or 24 h. (**B**) The ascorbate concentration of 25-day-old seedlings treated with either dosage (2 μmol·m^{-2}·s^{-1} or 4 μmol·m^{-2}·s^{-1}) of UV-B radiation at 4 h or 24 h. The plants without UV-B radiation served as controls. Three biologically independent replicate experiments were performed; data points represent the mean ± SE of three biological replicates. Asterisks indicate a significant difference (*P<0.05; **P<0.01) relative to the corresponding control, using Student's *t*-test.

Table S1. Gradient elution program for HPLC analysis.

Time (min)	Mobile phase A	Mobile phase B
0–8	95%–90%	5%–10%
8–15	90%–90%	10%–10%
15–26	90%–87%	10%–13%
26–35	87%–85%	13%–15%
35–40	85%–85%	15%–15%
40–45	85%–80%	15%–00%
45–55	80%–95%	15%–5%

Table S2. Primers used in quantitative reverse transcription PCR (qRT–PCR).

Gene	Forward primers (5'-3')	Reverse primers (5'-3')
BrActin2	ATGCTCCCAGGGCTGTTTTC	CCTCTTTTGGACTGTGCTTCG
BrPAL	ATGTTTCTTTCGTGCTGCCG	GGTTTTGGTGCTACTTCTCATCG
BrC4H	AGCCAACCTGCTCCTCTCTCC	AAGAACCCTGACTCCGCCAC
Br4CL	TGTGAAACATCGGGAGGAAAC	CGGGACTTCCAAAAGGTGTG
BrCHS	CAAAGAGGCGGCAGTGAAGG	AGACGCTTGACGGAAGGACG
BrCHI	CTTTCCCTCCTGGTGCTTCG	AATCTCTCGGCAAGACTCAACC
BrF3H	CTCACTTTAAGCGGATACACGG	CACTATCTGAGCAACGGGAGGT
BrF3′H	CGATGTGTGGTAACGAGAGTGG	CGGAGATAATGAGAAAAGCCC
BrFLS	ACATCGAAGGGTACGGAACG	TTAACGCACGACGGTGGC
BrDFR	CCAAGAAGCTGATTGATATGGG	CGGTAAAGTGAAGGGAGAAAACC
BrANS	GGTGGTTCACAAAACACAGCC	TGGAGATTCTTAGTAACGGGAGG
BrLDOX	TAGCACTTGGCGTGGAAGC	TGACCCATTTGCCCTCGTAG
BrUFGT	GTATCCGTTGCTCCTCCAGTG	GGGGAAGCCAATGAGACAGAAG
